# Forgiving, fast and slow: validity of the implicit association test for predicting differential response latencies in a transgression-recall paradigm

**DOI:** 10.3389/fpsyg.2014.00728

**Published:** 2014-07-11

**Authors:** Ramzi Fatfouta, Michela Schröder-Abé, Angela Merkl

**Affiliations:** ^1^Cluster of Excellence “Languages of Emotion,” Freie Universität BerlinBerlin, Germany; ^2^Affective Neuroscience and Psychology of Emotion, Department of Education and Psychology, Freie Universität BerlinBerlin, Germany; ^3^Department of Psychology, Technische Universität DarmstadtDarmstadt, Germany; ^4^Department of Neurology with Experimental Neurology, Charité–UniversitätsmedizinBerlin, Germany

**Keywords:** forgiveness, implicit self-concept, automaticity, Implicit Association Test (IAT), response latency measures

## Abstract

This study examined the role of automaticity in forgiving a real-life offense. As an alternative to self-report, an Implicit Association Test (IAT) of forgiveness was developed. Implicit (IAT-measured) and explicit (self-reported) forgiveness predicted shorter response times of state forgiveness ratings. The forgiveness IAT was highly reliable, moderately stable over time, and demonstrated incremental validity. Results suggest that the newly introduced forgiveness IAT could advance personality research beyond what is known from self-report measures, further corroborating the notion of implicit forgiveness. Implications for personality assessment are discussed.

## Introduction

Individual differences in forgiveness have traditionally been assessed via self-report. Contemporary measures, however, can be biased in two fundamental ways. First, they are susceptible to social desirability (Hoyt and McCullough, 2005), and second, they are insensitive to processes outside of awareness (Bornstein, 2002; Moors and De Houwer, 2006). This latter point is problematic as one might explicitly state one has forgiven an offense, yet implicitly continue harboring a grudge (hollow forgiveness; Fincham, 2010). To remedy these limitations, indirect measures may prove useful.

Indirect measures tap into different psychological processes than direct measures (i.e., self-reports). In accordance with dual-process models (for an overview, see Strack and Deutsch, 2004; Back et al., 2009), human behavior is a joint function of both reflective and impulsive processes. Reflective processes operate slowly and refer to propositional or explicit representations of the self that result from reasoning. Impulsive processes operate fast and refer to associative or implicit representations of the self that are activated automatically (i.e., non-deliberatively) when encountering situational cues. While reflective processes can be assessed via direct measures, impulsive processes can be assessed indirectly. Notably, indirect measures have been found to provide increments in predictive validity beyond direct measures—which highlights their value for a more complete assessment of trait factors and for the prediction of trait-relevant behavior (e.g., Back et al., 2009; Rudolph et al., 2010; Fleischhauer et al., 2013).

The Implicit Association Test (IAT; Greenwald et al., 1998) is the most popular indirect measure of automatic associations and has been shown to be relatively resistant to faking (Steffens, 2004; Röhner et al., 2011). Using a speeded categorization task, the IAT measures the (relative) association strength between a target concept (e.g., me–others) and an attribute dimension (e.g., forgiving–vengeful). The rationale is that when individuals strongly associate their self with congruent (e.g., me–forgiving) vs. incongruent (e.g., me–vengeful) attributes, categorization will be easier (i.e., faster). The IAT effect, defined as response-latency difference between congruent and incongruent pairings, thus reflects associative links between the self and a trait concept (in our case, forgiveness) and may be referred to as implicit self-concept of personality (Schnabel and Asendorpf, 2010). To date, however, no study has attempted to look at the implicit self-concept of forgiveness.

Existing research has conceptualized forgiveness as a deliberative process with a presumed endpoint, that is, one's decision to forgive a transgressor (Fincham, 2000; Fincham et al., 2005). Specifically, to forgive involves a prosocial change in individuals' thoughts, feelings, and motivations, whereby they become more positively disposed toward their transgressor (McCullough et al., 1998; Worthington and Wade, 1999). However, as Fincham et al. (2006, p. 422) noted, the focus on deliberative processes “may overlook aspects of forgiveness that occur outside of conscious awareness.” Indeed, recent work has provided empirical support for this contention (for an overview, see Karremans and Lange, 2008). In close relationships, for instance, the inclination to forgive occurs automatically and without the individuals' intention. The reasoning is that individuals automatically associate forgiveness with beneficial outcomes (e.g., relationship satisfaction), thereby reinforcing impulsive/automatic responding in a pro-relational manner (Karremans and Aarts, 2007, Studies 1 and 2). This is consistent with associative-learning processes in dual-process models (Strack and Deutsch, 2004; Back et al., 2009) and calls for a multimethod approach including indirect measures like the IAT. Consequently, it is necessary to examine both explicit *and* implicit aspects of forgiveness.

Based on these findings, the current study aimed to develop an IAT that taps individual differences in *implicit forgiveness* and to test its predictive validity over and above a corresponding self-report questionnaire. As forgiveness-related behavioral criteria, we used the response times (RTs) individuals need to rate their state forgiveness (i.e., their current thoughts and feelings toward an offender; McCullough et al., 1998). In past research, the measurement of response latency has been employed as an operative indicator of attitude strength (Fazio, 2001). The logic here is that RTs reflect the individual's mental effort to arrive at a response and therefore indicate the accessibility of information in memory. The more accessible the information is, the faster the individual responds (Fazio, 1990a,b). A personality-oriented adaptation of this approach has been provided by Lischetzke et al. (2005), who demonstrated that when individuals can easily access internal cues about their affective state they are faster to rate it.

With respect to forgiveness, a similar account has been proposed. Specifically, individuals who are consistently inclined to forgive are assumed to “have both a higher rate of forgiveness and a *shorter* latency of response” (Sutton and Thomas, 2006, p. 33; emphasis added). Hence, it appears plausible that when prompted (1) to recall an offense and then (2) to rate their state forgiveness, individuals who have already worked through the pain should more easily access internal cues about their forgiveness and, hence, exhibit faster responses. By contrast, individuals who have not yet achieved forgiveness (i.e., who find internal forgiveness cues to be rather difficult to access), should need comparatively more time to contemplate and report their responses. For example, responding to an item such as “I am finding it difficult to act warmly toward him/her” (McCullough et al., 2006) should be less effortful (i.e., RT should be faster) for a person who has come to terms with the hurt^1^.

Response latencies reflect both deliberative and automatic processing (Fazio, 1990a). Therefore, we hypothesized that both the newly developed forgiveness IAT and a forgiveness self-report would predict RT of forgiveness ratings. Specifically, high-forgiving individuals—measured by self-report and indirectly—should be faster to rate their state forgiveness (*Hypothesis 1*). Given that incremental validity is an important psychometric property when evaluating indirect measures (Perugini and Banse, 2007), we tested whether the novel forgiveness IAT predicted RT of state forgiveness ratings beyond explicit (self-reported) forgiveness (*Hypothesis 2*).

## Materials and methods

We report all data exclusions (if any), manipulations, and measures, and how we determined our sample size. The latter was determined a priori with the goal to obtain around 100 observations.

### Participants

One hundred and four students (70 women; *M*_age_ = 25.10, *SD* = 4.39) of Freie Universität Berlin participated in exchange for money or course credit.

### Procedure

Data were collected individually or in sessions of two individuals. After providing written informed consent, participants completed the forgiveness IAT and a set of questionnaires on a computer screen described below. Each questionnaire item appeared separately on the screen. Participants answered by mouse click on the respective response category. They had to click a *next* button to proceed with the next item.

At the beginning of the computer-based questionnaire, participants answered questions to obtain a measure of their baseline speed (see Baseline speed). Participants then had (1) to think of a close person; (2) type in his/her name; (3) indicate the type of relationship involved, and (4) rate their perception of closeness to the self-selected other (Karremans and Aarts, 2007). Next, participants were instructed to bring to mind a real-life situation in which the other had hurt them and to rate their state forgiveness. RT (i.e., the time between item presentation and click on the *next* button) was unobtrusively recorded (Lischetzke et al., 2005). Subsequently, participants rated the severity of the transgression and completed measures of explicit forgiveness as well as social desirability. Finally, participants were thanked and debriefed.

### Measures

#### Implicit forgiveness

Implicit forgiveness was measured with an IAT (Greenwald et al., 1998). The forgiveness IAT was specifically designed for this study and comprised five blocks (for procedural details, see Table 1). In each trial, a stimulus word was presented in the center of the screen. Participants then pressed either a left or a right key to categorize the stimulus as quickly and accurately as possible into one of the categories. The category labels appeared in the upper left and right hand corners of the screen.

**Table 1:** **Forgiveness IAT: Task Sequence and Stimuli**.

**Block**	**Task**	**Target concepts**	**Attribute concepts^a^**	**Key assignment^b^**	
				**Left (A)**	**Right (5)**
1 (20)	Target discrimination	Me–others		Me, my, own, I, self	They, your, them, you, others
2 (20)	Attribute discrimination		Forgiving–Vengeful	Forgiving, conciliatory, merciful, gracious, lenient	Vengeful, punitive, hostile, merciless, unforgiving
**3** (60)	Combined discrimination	Me–others	Forgiving–Vengeful	Me, my, own, I, self	They, your, them, you, others
				Forgiving, conciliatory, merciful, gracious, lenient	Vengeful, punitive, hostile, merciless, unforgiving
4 (40)	Reversed attribute discrimination		Vengeful–Forgiving	Vengeful, punitive, hostile, merciless, unforgiving	Forgiving, conciliatory, merciful, gracious, lenient
**5** (60)	Combined reversed discrimination	Me–others	Vengeful–Forgiving	Me, my, own, I, self	They, your, them, you, others
				Vengeful, punitive, hostile, merciless, unforgiving	Forgiving, conciliatory, merciful, gracious, lenient

*IAT, Implicit Association Test. The number of trials per block is in parentheses. Critical blocks are in bold*.

a *Stimuli were piloted (N = 50, M_age_ = 24.96, SD = 4.43) and matched according to familiarity, valence, similarity, and potency*.

b *Number 5 from the numeric keyboard*.

The first two blocks consisted of a simple discrimination task, in which participants practiced correctly categorizing stimuli from the target category (me–others) and attribute category (forgiving–vengeful). The third block combined both discrimination tasks (i.e., me–forgiving for the left key; others–vengeful for the right key). In block 4, the labels of the attribute categories were reversed (vengeful–forgiving). Block 5 consisted of the reversed combined discrimination task (i.e., me–vengeful for the left key; others–forgiving for the right key).

The order of critical blocks (i.e., blocks 3 and 5) was held constant to maximize the reliability of person effects (Banse, 2001). IAT-effects were computed using the improved scoring algorithm (D_1_; Greenwald et al., 2003). Higher scores reflect stronger automatic associations between me–forgiving (vs. me–vengeful), and hence, a more forgiving implicit self-concept.

To calculate the forgiveness IAT's reliability, we applied the D_1_–algorithm to two mutually exclusive subsets of the critical trials (Schmukle and Egloff, 2006). The Spearman-Brown corrected split-half correlation of this two-part measure was close to excellent (*r*_tt_ = 0.89). To provide an estimate of temporal stability, the last 30 participants (19 women; *M*_age_ = 26.60, *SD* = 4.35) were re-contacted after 1 month (*M*_time interval_ = 32.70 days, *SD* = 3.70) to complete the forgiveness IAT in a follow-up assessment. The temporal stability was moderate (0.50; 95% confidence interval = 0.34; 0.63) and similar to that previously reported for IATs (Egloff et al., 2005).

#### Baseline speed

Because we used RT as an individual difference measure, we controlled for differential baseline response speed (Fazio, 1990b). Baseline response speed was assessed by means of seven easy-knowledge questions [e.g., “What type of celestial body is the earth? (1) White giant; (2) Planet; (3) Asteroid; (4) Moon”; (Lischetzke et al., 2005)] to which the correct answer was evident, so that RT (α = 0.71) tapped only differential reading speed and motor behavior (e.g., muscle speed). Mean item accuracy was high (99.36%, *SD* = 0.03), thus corroborating this idea. Consistent with Lischetzke and colleagues, we omitted the first (training) item and calculated the median of the remaining six RT scores. Further validity evidence for this baseline speed measure can be found in Lischetzke et al. (2011).

#### Closeness

Closeness to the transgressor was assessed with the Inclusion of the Other in the Self scale (IOS; Aron et al., 1992), a pictorial measure comprising seven pairs of two increasingly overlapping circles, labeled “Self” and “Other.” Each pair was numbered and participants had to click on the respective response option to indicate their perceived closeness (1 = *not at all close*, 7 = *very close*). The IOS has demonstrated high alternate-form and test–retest stability as well as strong convergence with multi-item measures of relational closeness (Aron et al., 1992).

#### State forgiveness

State forgiveness was measured with the Transgression-Related Interpersonal Motivations Inventory (TRIM; McCullough et al., 1998, 2006). It assesses participants' motivational changes toward a specific transgressor and is divided into three subscales, with five items measuring revenge (e.g., “I'm going to get even”), seven items measuring avoidance (e.g., “I keep as much distance between us as possible”), and six items measuring benevolence (“I have given up my hurt and resentment”). Items were rated on a 7-point Likert scale (1 = *strongly disagree*, 7 = *strongly agree*). Past research has evidenced that the subscales have high internal consistency, moderate temporal stability, and evidence of construct validity (McCullough et al., 1998, 2006; McCullough and Hoyt, 2002). In this sample, internal consistency (α) was good for avoidance and benevolence (0.87 and 0.76, respectively), but somewhat modest for revenge (0.53). While this alpha is less than ideal, similar estimates have been reported with the revenge scale (α = 0.52; Allemand et al., 2013). Unlike previous research, however, our primary analyses focused on participants' RTs, and hence, this value is deemed reasonable.

#### RT of state forgiveness ratings

As an objective measure of state forgiveness, RT to each TRIM item was recorded. Again, the first item was regarded as a training item and its RT was excluded from the analyses. We then log-transformed (log_e_[x]) RTs for each person to correct for positive skewness (Fazio, 1990b) and calculated mean RT scores for each of the three TRIM subscales: RT_Revenge_(α = 0.65), RT_Avoidance_(α = 0.78), and RT_Benevolence_(α = 0.70). Using three regression equations, we computed—separately for each TRIM subscale—residual RT scores by partialling out baseline speed. Values greater (smaller) than zero indicate that an individual is slower (faster) than predicted by his or her baseline speed. Specifically, the residual (i.e., baseline-corrected) RT scores reflect the speed with which individuals rate their revenge, avoidance, and benevolence, respectively.

#### Transgression severity

On completion of the TRIM, participants indicated how severe they perceived their transgression to be on a face-valid, single-item scale (Ghaemmaghami et al., 2011). Specifically, they were asked: “How painful was the transgression to you at the time it occurred?” Responses were made on a 7-point scale (1 = *not painful at all, 7 = very painful*).

#### Explicit forgiveness

Explicit forgiveness was measured with the four-item Tendency to Forgive Scale (TTF; Brown, 2003; e.g., “I tend to get over it quickly when someone hurts my feelings,” α = 0.76). Participants responded to each item on a 7–point Likert scale (1 = *strongly disagree*, 7 = *strongly agree*). The TTF has consistently demonstrated good psychometric properties, including strong predictive validity across several studies (Brown, 2003; Brown and Phillips, 2005).

#### Social desirability

The Balanced Inventory of Desirable Responding (BIDR; Paulhus, 1994; German version by Musch et al., 2002) was used to assess both factors of social desirability, impression management (10 items; e.g., “I never swear,” α = 0.76) and self-deceptive enhancement (10 items; e.g., “My first impressions of people usually turn out to be right,” α = 0.62). Participants rated each item on a 7–point Likert scale (1 = *strongly disagree*, 7 = *strongly agree*). We computed continuous (vs. dichotomized) subscale scores due to their superior psychometric properties (Stöber et al., 2002). The BIDR is widely used and both subscales have provided evidence of reliability and validity in past research (Paulhus, 1984, 1994; Musch et al., 2002).

### Statistical analyses

We analyzed bivariate correlations and conducted hierarchical multiple regression analyses using implicit and explicit forgiveness to predict residual (i.e., baseline-corrected) RT scores (i.e., RT_Revenge_, RT_Avoidance_, and RT_Benevolence_). All statistical tests adopted a significance level of α = 0.05 (two-tailed).

## Results

### Descriptive statistics and correlations among measures

Participants reported transgressions pertaining to romantic partners (38.5%), close friends (37.5%), parents (10.6%), other family members or relatives (10.6%), coworker, acquaintance or neighbor (1.0%), or other person (1.9%). Table 2 details descriptive statistics for all measures.

**Table 2:** **Means, Standard Deviations, and Actual Ranges of all Study Variables**.

**Variable**	***M***	***SD***	**Min**	**Max**
**BASELINE SPEED (S)^a^**				
RT to knowledge items	5.87	1.40	3.55	10.02
**TRAIT FORGIVENESS**				
Forgiveness IAT (D_1_)	0.47	0.25	−0.20	1.03
TTF	3.79	1.24	1.25	6.75
**STATE FORGIVENESS (TRIM)**				
Revenge	1.66	0.66	1.00	3.80
Avoidance	2.36	1.12	1.00	5.57
Benevolence	5.89	0.76	3.50	7.00
**RT OF STATE FORGIVENESS RATINGS (S)^a^**				
RT_Revenge_	4.89	1.37	3.00	8.75
RT_Avoidance_	4.57	1.11	2.81	8.06
RT_Benevolence_	5.68	1.32	3.48	9.49
**TRANSGRESSION-RELATED CHARACTERISTICS^b^**				
Closeness (IOS)	5.23	1.17	3.00	7.00
Transgression severity	4.93	1.65	1.00	7.00
**SOCIAL DESIRABILITY (BIDR)**				
Self-deceptive enhancement	4.05	0.72	2.50	6.00
Impression management	3.75	0.88	1.60	6.20

*IAT, Implicit Association Test; TTF, Tendency to Forgive Scale; TRIM, Transgression-Related Interpersonal Motivations Inventory; RT, response time; IOS, Inclusion of the Other in the Self Scale; BIDR, Balanced Inventory of Desirable Responding; Possible range of self-report scores: 1–7*.

a *For clarity of presentation, these values represent raw (mean) latencies*.

b *Single-item measures*.

Intercorrelations are presented in Table 3. Implicit and explicit forgiveness were not significantly correlated, a result that parallels findings on implicit-explicit relations in other research domains such as self-esteem (Bosson et al., 2000). Importantly, implicit forgiveness was unrelated to both social- desirability components. Explicit forgiveness, by contrast, correlated significantly positively with self-deceptive enhancement.

**Table 3:** **Intercorrelations Among Study Variables**.

**Variable**	**1**	**2**	**3**	**4**	**5**	**6**	**7**	**8**	**9**	**10**	**11**	**12**
**TRAIT FORGIVENESS**												
1. Forgiveness IAT (D_1_)	–											
2. TTF	0.00	–										
**STATE FORGIVENESS (TRIM)**												
3. Revenge	−0.04	−0.29^**^	–									
4. Avoidance	−0.05	−0.26^**^	0.37^***^	–								
5. Benevolence	0.01	0.28^**^	−0.37^***^	−0.51^***^	–							
**RESIDUAL RT OF STATE FORGIVENESS RATINGS**^a^												
6. Residual RT_Revenge_	−0.22^*^	−0.24^*^	0.36^***^	0.22^*^	−0.07	–						
7. Residual RT_Avoidance_	0.01	−0.17^#^	0.12	0.40^***^	−0.15	0.58^***^	–					
8. Residual RT_Benevolence_	−0.18^#^	−0.27^**^	−0.02	0.10	−0.02	0.43^***^	0.54^***^	–				
**TRANSGRESSION-RELATED CHARACTERISTICS**^b^												
9. Closeness (IOS)	−0.11	−0.02	−0.17^#^	−0.20^*^	0.25^*^	−0.16	−0.15	−0.10	–			
10. Transgression severity	−0.18^#^	−0.16	−0.09	−0.05	−0.12	0.14	0.02	0.02	0.04	–		
**SOCIAL DESIRABILITY (BIDR)**												
11. Self-deceptive enhancement	−0.10	0.23^*^	0.03	−0.01	0.06	−0.04	−0.05	−0.07	0.14	−0.07	–	
12. Impression management	−0.04	0.02	−0.08	−0.19^#^	0.09	0.00	−0.01	0.00	0.14	0.01	0.15	–

IAT, Implicit Association Test; TTF, Tendency to Forgive Scale; TRIM, Transgression-Related Interpersonal Motivations Inventory; RT, response time; IOS, Inclusion of the Other in the Self Scale; BIDR, Balanced Inventory of Desirable Responding; Possible range of self-report scores: 1–7.

a Baseline speed partialled out.

b *Single-item measures*.

# *p < 0.10*.

* *p < 0.05*.

** *p < 0.01*.

*** *p < 0.001*.

### Predicting RT from implicit and explicit forgiveness

Our main predictions were that both implicit and explicit forgiveness would predict shorter RT of forgiveness ratings (*Hypothesis 1*) and that implicit forgiveness would demonstrate increments in predictive validity (*Hypothesis 2*). To test these hypotheses, we computed three hierarchical multiple regression analyses. Each residual RT score (i.e., RT_Revenge_, RT_Avoidance_, RT_Benevolence_) was treated as a separate criterion and the trait forgiveness measures were entered in two steps (Step 1: explicit forgiveness; Step 2: implicit forgiveness). As such, we were able to estimate the incremental proportion of variance in residual RT accounted for by implicit forgiveness, after controlling for the effect of explicit forgiveness. We obtained qualitatively identical results when controlling for closeness and transgression severity in the first step of the models. Also, the interaction between implicit and explicit forgiveness yielded no significant effects. In the interest of parsimony, these variables were trimmed from the final analyses. Table 4 details the results.

**Table 4:** **Hierarchical Multiple Regression Analyses Predicting Response Time by Implicit and Explicit Forgiveness**.

**Residual RT of State Forgiveness Ratings^a^**						
**Predictor**	**RT^b^_Revenge_**		**RT^c^_Avoidance_**		**RT^d^_Benevolence_**	
	**ΔR^2^**	**β**	**ΔR^2^**	**β**	**ΔR^2^**	**β**
Step 1	0.060^*^		0.029^#^		0.071^**^	
TTF		−0.24^*^		−0.17^#^		−0.27^**^
Step 2	0.047^*^		0.000		0.031^#^	
TTF		−0.24^*^		−0.17^#^		−0.27^**^
Forgiveness IAT		−0.22^*^		0.01		−0.18^#^

*RT, response time; TTF, Tendency to Forgive Scale; IAT, Implicit Association Test; β, standardized beta coefficient; ΔR^2^, change in explained variance from one step to the next*.

a *Baseline speed partialled out*.

b Step 1: F_(1, 102)_ = 6.46, p = 0.013; Step 2: F_(2, 101)_ = 6.05, p = 0.003;

c Step 1: F_(1, 102)_ = 3.03, p = 0.085; Step 2: F_(2, 101)_ = 1.50, p = 0.227;

d *Step 1: F_(1, 102)_ = *7.78*, p = 0.006; Step 2: F_(2, 101)_ = 5.71, p = 0.004*.

# *p < 0.10*.

* *p < 0.05*.

** *p < 0.01*.

The first regression model revealed that both implicit and explicit forgiveness significantly predicted residual RT_Revenge_. As expected, individuals high in implicit and explicit forgiveness were faster to rate their revenge motivations. Introducing implicit forgiveness in Step 2 added significantly to the prediction of residual RT_Revenge_, over and above explicit forgiveness. The full model explained 10.7% of variance in residual RT_Revenge_.

The second regression model with residual RT_Avoidance_ as criterion revealed that explicit forgiveness predicted the time individuals needed to rate their avoidance motivations, but implicit forgiveness did not. Consequently, the addition of implicit forgiveness in Step 2 explained no variation in residual RT_Avoidance_. The restricted model (i.e., the model containing only explicit forgiveness as predictor) explained a small (2.9%) and marginally significant proportion of variance in the RT measure.

The model predicting residual RT_Benevolence_ was examined last. As expected, high levels of implicit and explicit forgiveness were associated with shorter RT for items tapping benevolence. The addition of implicit forgiveness in Step 2, however, contributed only a marginally significant amount of variance to the prediction of residual RT_Benevolence_, beyond explicit forgiveness. The full model explained 10.2% of variance in residual RT_Benevolence_.

In summary, *Hypothesis 1* was generally supported for explicit forgiveness: among individuals high in explicit forgiveness, RT was shorter for all three TRIM dimensions (i.e., revenge, avoidance, benevolence) and significantly so for revenge and benevolence. As regards implicit forgiveness, *Hypotheses 1* and *2* were supported for one of the three RT scores (i.e., residual RT_Revenge_), providing suggestive evidence for the indirect forgiveness measure in terms of incremental predictive validity.

## Discussion

In the present study, we analyzed the predictive and incremental validity of a non-self-report (indirect) measure of forgiveness, the forgiveness IAT. As forgiveness-related behavioral criteria, we examined differential response latencies of state forgiveness ratings.

The new forgiveness IAT provides an indirect assessment of forgiveness by measuring chronically accessible self-associations pertaining to forgiving vs. vengeful. Noteworthy, we found that the IAT and the TTF scale were virtually unrelated. This may indicate that both measures tap different modes of representation (i.e., automatic vs. controlled; Moors and De Houwer, 2006) and different content (i.e., the stimuli included in both instruments). Notably, such factors (i.e., measurement correspondence and processing mode) have been shown to reduce the correlation between direct and indirect measures (Hofmann et al., 2005).

As desired for personality assessment, the forgiveness IAT demonstrated high split-half reliability, comparable with coefficients in related research on the implicit personality self-concept (Egloff and Schmukle, 2002; Back et al., 2009) and similar to those of explicit forgiveness measures (Brown, 2003; Brown and Phillips, 2005). Concerning temporal stability (i.e., test–retest reliability), IAT scores demonstrated a moderate convergence across measurement occasions. The 1-month retest correlation in the current study (*r* = 0.50) was in the range typically found for IATs (*r* = 0.25−0.69, with mean and median estimates of 0.50; Lane et al., 2007). Given the small test–retest sample size, however, this estimate should be viewed with caution.

As to the prediction of behavior, results demonstrated that among individuals high in explicit forgiveness, RT was shorter for two of the three TRIM dimensions (i.e., revenge and benevolence). For implicit forgiveness, hypotheses were supported for one of the three RT scores (i.e., residual RT_Revenge_). Lack of conceptual correspondence (Ajzen and Fishbein, 1977) might explain why implicit forgiveness did not predict residual RT_Avoidance_, given that the concept of avoidance is not directly tapped with our IAT.

Overall, our findings are consistent with well-established evidence that indirect personality measures allow prediction of variance in behavior beyond what is already predicted by self-reports (Back et al., 2009; Greenwald et al., 2009). Although not significantly related to TTF scores, the forgiveness IAT provided incremental insights into trait-relevant criteria that would have been overlooked if our focus had been restricted to self-report. This parallels recent results from the fields of need for cognition or self-esteem (Rudolph et al., 2010; Fleischhauer et al., 2013), which demonstrated that indirect measures (although not significantly associated with direct measures) incrementally explained variance in relevant behavior.

Apart from those results, some methodological considerations need to be discussed. For example, our criterion measures were based on response latencies and not on *actual* forgiving behavior. Although important to mention, prior personality research using the IAT similarly operationalized behavior with RTs. In one study on implicit self-esteem, for example, the time individuals spent reading social feedback has been taken as an objective measure of defensiveness (Schröder-Abé et al., 2007; study 2). Additionally, unlike other personality traits (e.g., neuroticism) that are comparatively easy to observe (e.g., non-verbal nervousness; Back et al., 2009), forgiveness is an intrinsically processual phenomenon that unfolds over time (McCullough et al., 2003), thus not manifested in a specific act (Fincham, 2000). Therefore, actual behavior might be less suited for further research on implicit forgiveness.

It might be valuable, however, to examine other indices, such as physiological reactivity. Johnston et al. (2013), for instance, have recently demonstrated that implicit—but not explicit—moral identity predicted increases in blood pressure and heart rate in response to moral transgressions. Given evidence that the same physiological indicants are associated with unforgiving responses (Witvliet et al., 2001), one testable prediction is that the forgiveness IAT should predict individual variation in physiological responses to *personal* transgressions.

Together, our research has important implications for personality theory and assessment. First, it suggests that an individual's implicit cognition may influence the way he or she responds to transgression. This resonates with recent work on the role of implicit processes in forgiveness (Karremans and Aarts, 2007). Second, our findings provide an example of how direct and indirect measures complement each other in targeting implicit *and* explicit personality features, thereby yielding a more comprehensive understanding of behavior (Bornstein, 2002). Future research needs to place the construct of *implicit forgiveness* within a broader nomological network, further establishing its construct validity with respect to related constructs (e.g., empathy for the offender). Third, the IAT's resistance to social desirability underscores its utility for assessing even constructs as desirable as forgiveness. Last, the results of this study demonstrate that implicit associations may contribute a valuable, albeit understudied, source of information in understanding the implicit self-concept of forgiveness. Therefore, we emphasize the importance of administering multiple assessment formats, especially in applied (e.g., clinical) contexts. Promising work has already begun on the differential treatment sensitivity of implicit (vs. explicit) personality measures (Gamer et al., 2008) and should be further explored.

## Conclusion

The present study developed an indirect measure of forgiveness—the forgiveness IAT—and examined its incremental validity for the prediction of differential response latencies in a transgression-recall paradigm. We thereby complement previous work on the role of automatic processes in forgiveness and, importantly, provide an addition to existing self-reports. We found preliminary evidence that implicit forgiveness explained additional variance above and beyond a corresponding explicit measure. Together, our findings provide novel insights that will hopefully stimulate both research and practice of implicit personality functioning.

### Conflict of interest statement

The authors declare that the research was conducted in the absence of any commercial or financial relationships that could be construed as a potential conflict of interest.

## Footnotes

^1^To demonstrate that higher forgiveness is indeed related to faster responses, we performed a pilot study (*N* = 120, *M*_age_ = 22.47, *SD* = 4.33), instructing participants to recall an experienced hurt and to rate their motivation to seek revenge against the transgressor (while RT was unobtrusively measured). Participants then indicated their forgiveness (i.e., “I have forgiven what he/she did to me”). As predicted, RT was significantly negatively correlated with forgiveness (*r* = −0.20, *p* = 0.031).
